# Ethnobotanical investigation of traditional medicinal plants commercialized in the markets of Mashhad, Iran

**Published:** 2013

**Authors:** Mohammad Sadegh Amiri, Mohammad Reza Joharchi

**Affiliations:** 1 *Department of Biology, Payame Noor University, Tehran, I. R. Iran*; 2 *Research Center for Plant Sciences, Ferdowsi University of Mashhad, Mashhad, I. R. Iran*

**Keywords:** Ethnobotany, Iran, Market, Mashhad, Medicinal Plants

## Abstract

**Objective:** An ethnobotanical survey on the medicinal plant species marketed in Mashhad city, northeastern Iran, was conducted in order to document traditional medicinal knowledge and application of medicinal plants.

**Materials and Methods:** This study was undertaken between 2011 and 2012. The indigenous knowledge of traditional healers used for medicinal purposes were collected through questionnaire and personal interviews during field trips. Ethnobotanical data was arranged alphabetically by family name followed by botanical name, vernacular name, part used, folk use, and recipe. Correct identification was made with the help of the various Floras and different herbal literature at the Ferdowsi University of Mashhad Herbarium (FUMH).

**Results:** The present investigation reported medicinal information for about 269 species, belonging to 87 vascular plant families and one fungus family. The most important family was Lamiaceae with 26 species, followed by Asteraceae with 23, Fabaceae with 20, and Apiaceae with 19. Herbal medicine uses reported by herbalists was classified into 132 different uses which show significant results to treat a wide spectrum of human ailments. Plants sold at the market were mostly used for digestive system disorders, respiratory problems, urological troubles, nervous system disorders, skin problems, and gynecological ailments.

**Conclusion:** This survey showed that although people in study area have access to modern medical facilities, a lot of them still continue to depend on medicinal plants for the treatment of healthcare problems. The present paper represents significant ethnobotanical information on medical plants which provides baseline data for future pharmacological and phytochemical studies.

## Introduction

The usage of medicinal plants presents a very important aspect of the traditional medicine which is imbedded in the culture of people of developing countries (Kloucek et al., 2005 ▶). In many developing countries, medicinal plants have not been well studied, tested, or documented. Most of the information is still in the hands of traditional healers and knowledge of healers is either lost or passed to next generation by the word of mouth (Yirga, 2010 ▶). The extent of the knowledge of traditional medicine practice based on medicinal plants should be documented through botanical surveys. Botanical collection and documentation of the associated ethno-botanical knowledge should be carried out before such rich heritages are lost due to various anthropogenic and other natural causes (Martin, 1995 ▶).

 Traditional herbal medicine has played an important role in Iran. Iran has a very honorable past in traditional medicine, which goes back to the time of Babylonian-Assyrian civilization. One of the most significant ancient heritages is sophisticated experience of people who have tried over the millennia to discover useful plants for health improvement and each generation added their own experience to this tradition (Naghibi et al., 2005 ▶). Today, medicinal plants are still widely used in Iran. In all cities and villages, there are specific stores (named Attari), which traditional healers (Attar) give receipts and sell medicinal plants. People use medicinal plants as curatives or palliatives of main health problems according to their cultural background. The notable use and commercialization of medicinal plants to alleviate and cure health problems and ailments in all cities of the country, points out the importance of these natural resources in the folk medicine and culture of the Iranian people (Emami et al., 2012 ▶). Despite the vast knowledge of medicinal plants existed in Iran, a few attempts have been carried out to document ethnobotanical knowledge. Some researchers have investigating the traditional pharmacopoeia and medicinal plants in different areas of the country (Amin, 2006 ▶; Amiri, 2012 ▶; Emami et al., 2012 ▶; Ghorbani, 2005 ▶; Hooper and Field, 1937 ▶; Miraldi et al., 2001 ▶; Mosaddegh et al., 2012 ▶; Naghibi et al., 2005 ▶; Rajaei, 2012 ▶; Safa et al., 2013 ▶; Zargari, 1989-1992 ▶;). 

The objective of this work was to elicit data on the traditional uses of medicinal plants marketed in Mashhad city and preserve it to be used by the next generations. In addition, the present paper provides baseline data for future pharmacological and phytochemical studies.

## Materials and Methods

**Study area**

Mashhad is located in the northeast of Iran. It is the second largest city in Iran and one of the holiest cities in the world. Its approximate geographic location is to  north latitude and to  east longitude, in the valley of the Kashaf River near Turkmenistan, between the two mountain ranges of Binalood and Hezar-masjed. It is located in the center of the Razavi Khorasan Province close to the borders of Afghanistan and Turkmenistan. The total area of the Mashhad is 270 km^2^ and the population of the city is about 3 million people. There are also over 20 million pilgrims who visit the city every year. 

The vast majority of the Mashhad people are ethnic Persians who form over 95% of the city's population. Other ethnic groups include Kurdish and Turkmen people who have immigrated recently to the city from the North Khorasan province. Among the non-Iranians, there are small immigrants from Afghanistan, Iraq, and Pakistan.

## Methods

In order to gather information on medicinal species that were found in the markets of Mashhad, a survey was carried out during the years 2011-2012. Ethnobotanical interview was used as the basis for data gathering. A questionnaire was administered only to people who had knowledge of medicinal plants, through face to face interviews. Totally, more than 100 informants (Attar) with in the age of 37 to 82 were interviewed; these included males and females. Ethnobotanical information, including the various data such as name and age of informants, local names, purpose of usage, preparation procedure, and duration of the treatment were obtained through interviews and discussions. After collecting the specimens, we represented these specimens to different people to confirm the accuracy of the results. Subsequently, specimens of the reported medicinal plants were identified by specialist with the help of available Floras (Rechinger, 1963-2005 ▶; Assadi et al., 1988-2008 ▶) and consulting with different herbal literature (Amin, 1991 ▶; Hooper, 1937 ▶; Zargari, 1989-1992 ▶) at the Ferdowsi University of Mashhad Herbarium (FUMH). In this paper, scientific and author names of plant species were checked for accuracy according to the plant list (www.theplantlist.org).

## Results

Markets have long been recognized as places that reflect regional trade and culture, and have been used to study the commercialization and utilization of natural products (Hooper and Field, 1937 ▶). During the present study, it was observed that 269 medicinal plant species distributed in 224 genera, belonging to 88 different families were sold in the markets of Mashhad which were used by the local inhabitants for curing various diseases. From the point of view of Taxonomy, plants that are being used for therapeutic purpose in this region belong to divisions of Pteridophyta (one species from Equisetaceae, Polypodiaceae, and Pteridaceae) and Spermatophyta with its two subdivisions: Coniferophytina (one species from Cupressaceae, Ephedraceae, and Pinaceae) and Magnoliophytina (with two classes Magnoliopsida and Liliopsida, including 81 families all together). Domination of Magnoliopsida has been very noticeable with its 69 families comprised of 232 species that are used in therapeutic purposes. Maximum number of medicinal plant species belongs to family Lamiaceae (26 species) followed by Asteraceae (23 species), Fabaceae (20 species), and Apiaceae (19 species). Class Liliopsida is represented by twelve families which the most important family was Zingiberaceae with 7 species, followed by Orchidaceae, Poaceae with 4 and Amaryllidaceae, Asparagaceae each with 3 species. The rest of the families are represented by one or two species only. 

According to Table 1, *Astragalus *belonging to family Fabaceae was found to be the largest genus in the market samples in the research area. The most important species of* Astragalus *which produce katira is *Astragalus* *gummifer* Labill and this popular herbal drug can also be obtained from other species of this genus (i.e.,* Astragalus cerasocrena*,* Astragalus echidna*,* Astragalus floccosus*, *Astragalus eriosphaerus*, *Astragalus hypsogeton*,* Astragalus turkmenorum, *and* Astragalus verus*). The plants used for medicinal purposes in the various markets of Mashhad are arranged in alphabetical order of their family and botanical names, with the relevant information (Table 1).

**Table 1:** Medicinal plants used by the traditional healers for the treatment of various ailments in the markets of Mashhad, Iran.

**NO**	**Family**	**Scientific name**	**Local name**	**Part used**	**Status**	**Medicinal** ** uses**
1	Acoraceae	*Acorus* *calamus* L.	Agir Torki	Root	Im^1^	Treatment of Urinary Incontinence, Diuretic, Carminative, Stimulant, Hematinic
2	Amaranthaceae	*Amaranthus caudatus *L.	Taj Khorus	Aerial parts	In^1^	Disinfectant Treatment of Enteritis, Febrifuge, Antitussive, Antidiarrhea, Laxative,
3	Amaranthaceae	*Dysphania botrys *(L.) Mosyakin & Clemants	Dermaneh Torki	Aerial parts	In^1^	Diabetes, Treatment of Sinusitis, Respiratory disorders, Anthelmintic, Antacid, Antidiarrhea, Carminative, Urinary Antiseptic
4	Amaryllidaceae	*Allium altissimum* Regel	Musir	Bulb	In^1^	Antiseptic, Appetizer, Digestive
5	Amaryllidaceae	*Allium* *cepa *L*.*	Piaz	Seed	In^1^	Treatment of Trichoptlosis
6	Amaryllidaceae	*Allium* *sativum* L*.*	Sir	Bulb	In^1^	Hypoglycemic, Cardiac Diseases, Antiseptic, Toothache, Antihyperlipidemia, Anthelmintic, Antihypertensive
7	Anacardiaceae	*Mangifera indica* L	Anbeh	Leaves	Im^1^	Lung Cancer
8	Anacardiaceae	*Pistacia* *atlantica* Desf. ssp. *kurdica*	Saghez	Oleoresin	In^1^	Appetizer, Digestive, Antacid,
9	Anacardiaceae	*Pistacia* *atlantica* Desf. ssp*. mutica*	Baneh	Fruit	In^1^	Laxative, Tonic Stimulant,Treatment of Anaemia
10	Anacardiaceae	Pistacia lentiscus L.	Mastaki	Gum	Im^1^	Strengthening of Memory, Strengthening of Teeth Gum, Antihalitosis
11	Anacardiaceae	*Rhus* *coriaria* L*.*	Somagh	Fruit	In^1^	Jaundice, Cholesterol Lowering, Diabetes, Antihypertensive, Antidiarrhea, Anti-hemorrhage, Flavoring
12	Anacardiaceae	*Semecarpus* *anacardium* L*.*f.	Belador	Fruit	Im^1^	Tonic, Removal of Bad Foot's Odour
13	Apiaceae	*Anethum* *graveolens* L.	Shevid	Fruit	In^1^	Abortion, Anti-Dysmenorrhea, Galactogogue, Antihyperlipidemia, Carminative
14	Apiaceae	*Apium* *graveolens* L.	Karafs	Fruit	In^1^	Emmenagogue, Diuretic, Carminative
15	Apiaceae	*Bunium cylindricum* (Boiss. & Hohen.) Drude	Zireh Siah	Fruit	In^2^	Carminative
16	Apiaceae	Bunium persicum (Boiss.) B.Fedtsch.	Zireh Siah	Fruit	In^1^	Obesity, Galactogogue, Flavoring , Carminative, Calmative, Appetizer, Indigestion
17	Apiaceae	*Coriandrum* *sativum* L.	Geshniz	Fruit	In^1^	Acne, Treatment of Flatulence, Appetizer, Aphrodisiac, Calmative, Jaundice, Antiseptic, Aromatic
18	Apiaceae	*Conium* *maculatum* L.	Shokaran	Root	In^1^	Cholagogue**,** Depilator, Treatment of Dermal Allergies
19	Apiaceae	*Cuminum* *cyminum* L.	Zireh Sabz (Keravieh)	Fruit	In^1^	Treatment of Colic, Galactogogue, Obesity, Digestive, Favoring, Antiasetic
20	Apiaceae	*Daucus* *carota* L.	Havij	Fruit	In^1^	Diuretic, Emmenagogue
21	Apiaceae	*Dorema* *ammoniacum* D.Don	Kandal	Gum- Root	In^1^	Cystitis, Digestive, Treatment of Colic, Treatment of Furuncles, Expectorant, Anthelmintic, Emmenagogue, Anticovulsion
22	Apiaceae	*Falcaria* *vulgaris* Bernh.	Ghaz Yaghi	Leaves- Fruit	In^1^	Treatment of Vitiligo, Cut, Wound
23	Apiaceae	*Foeniculum* *vulgare* Mill.	Razianeh	Fruit	In^1^	Galactogogue, Digestive, Bronchitis, Appetizer, Antacid, Flatulence
24	Apiaceae	*Ferula* * foetida* (Bunge) Regel	Anghuzeh	Gum	In^1^	Anthelmintic, Treatment of Colic, Emmenagogue
25	Apiaceae	*Ferula* *gummosa* Boiss.	Barijeh	Gum- Root	In^1^	Anthelmintic Anticatarrhal, Antiallergic, Dyspepsia, Appetizer, Emmenagogue
26	Apiaceae	*Heracleum* *persicum* Desf.	Golpar	Fruit	In^1^	Treatment of Hiccup, Appetizer, Flavoring, Carminative, Anthelmintic, Stomach Tonic
27	Apiaceae	Levisticum officinale W.D.J.Koch	Angedane roomi	Fruit	In^1^	Nerve Diseases, Heart Tonic, Indigestion
28	Apiaceae	Petroselinum crispum (Mill.) Nyman ex A. W. Hill	Jafari	Fruit	In^1^	Emmenagogue, Diuretic, Carminative, Kidney Disorders
29	Apiaceae	*Pimpinella* *anisum* L.	Anison (Badian roomi)	Fruit	In^1^	Treatment of Flatulence, Anthelmintic,Treatment of Colic, Antacid, Stomachache, Antidiarrhea
30	Apiaceae	*Trachyspermum* *ammi *(L.) Sprague	Zenyan (Khordaneh)	Fruit	In^1^	Carminative, Anthelmintic, Antidiarrhea, Treatment of Colic, Antacid, Galactogogue
31	Apiaceae	*Zosima orientalis *Hoffm.	Angedane roomi	Fruit	In^2^	Nerve Diseases, Indigestion
32	Araliaceae	Panax ginseng C.A.Mey.	Jin Sing	Root	Im^1^	Aphrodisiac, Nerve Tonic, Treatment of Dyspepsia
33	Arecaceae	*Areca* *catechu* L.	Fufal	Seed	Im^1^	Treatment of premature Ejaculation, Aphrodisiac,Tonic, Anti-hemorrhage, Antidiarrhea, Treatment of Postpartum Bleeding, Stomach Tonic
34	Arecaceae	*Cocos* *nucifera* L.	Nargil	Fruit	Im^1^	Tonic, Hair Tonic, Treatment of Anaemia
35	Aristolochiaceae	*Aristolochia* *rotunda* L.	Zaravand	Root	Im^1^	Emmenagogue, Diuretic, Anti-Atherosclerosis, Tonic, Treatment of Rheumatism
36	Aristolochiaceae	*Asarum* *europaeum* L.	Asarun	Root	Im^1^	Tonic, Stimulant
37	Asparagaceae	*Aloe* *vera* (L.) Burm.f.	Sabre Zard	Leaves- Latex	Im^1^	Obesity, Antihemorrhoids, Purgative, Antihistaminic,Treatment of burn
38	Asparagaceae	Drimia *maritima* (L.) Stearn	Onsol	Bulb	In^1^	Arthrodynia, Emmenagogue, Hair Tonic
39	Asparagaceae	Polygonatum orientale Desf.	Shaghaghol	Root	In^1^	Tonic, Diuretic, Nerve Tonic, Aphrodisiac
40	Asteraceae	*Achillea santolinoides subsp. wilhelmsii *(K.Koch) Gruter	Bumadaran	Aerial parts	In^1^	Antihemorrhoids, Antidiarrhea, Hypoglycemic, Anthelmintic, Mastitis, Antacid, Dyspepsia, Nerve Tonic, Treatment of Osteoarthritis, Treatment of Blood Flooding, Appetizer
41	Asteraceae	*Anacyclus pyrethrum *(L.) Lag.	Aghergherha	Root	Im^1^	Treatment of Sciatica, Treatment of Stuttering
42	Asteraceae	*Arctium* *lappa* L.	Baba Adam	Leaves- Root	In^1^	Duretic Cholagogue, Depurative, Hypoglycemic,
43	Asteraceae	*Artemisia* *absinthium *L.	Afsantin	Aerial parts	In^1^	Anthelmintic, Appetizer, Indigestion
44	Asteraceae	*Artemisia* *dracunculus* L.	Tarkhun	Leaves	In^1^	Appetizer, Dyspepsia , Anthelmintic, Antacid, Carminative
45	Asteraceae	Artemisia vulgaris L.	Baranjasef	Flower	In^1^	Nerve Tonic, Sexual Impotency, Menstrual Regulator
46	Asteraceae	*Calendula* *officinalis* L.	Hamishe Bahar	Flower	Im^1^	Eczema, Treatmen of Dermal Disorders, Sudorific, Blood cleanser
47	Asteraceae	*Carthamus* *tinctorius* L.	Golrang (kajireh)	Flower- Seed	In^1^	Emmenagogue, Flavoring Luxative, Treatment of Rheumatism,
48	Asteraceae	*Centaurea* *behen* L.	Bahman Sefid	Root	In^1^	Aphrodisiac, Anti-lithiasis
49	Asteraceae	Centaurea depressa M.Bieb.	Gole Gandom	Aerial parts	In^1^	Digestive, Febrifuge, Cholagogue, Blood Cleanser, Antigout
50	Asteraceae	*Cichorium* *intybus* L*.*	Kasni	Aerial parts	In^1^	Treatment of Palpitation, Appetizer, Depurative, Treatment of Furuncles, Jaundice, Febrifuge, Antiallergic
51	Asteraceae	*Cynara* *scolymus* L.	Kangar Farangi	Aerial parts	Im^1^	Liver Tonic, Digestive, Jaundice, Hepatitis
52	Asteraceae	*Doronicum* *pardalianches* L.	Daroanj Aghrabi	Root	Im^1^	Diuretic, Treatment of Snake and Scorpion bites, Nerve Tonic
53	Asteraceae	*Echinops* *cephalotes *DC.	Shekar Tighal	Manna	In^1^	Antitussive, Anti-asthmatic, Pharyngitis, Febrifuge
54	Asteraceae	*Gundelia* *tournefortii* L*.*	Kangar	Aerial parts	In^1^	Liver Tonic Treatment of Hepatitis
55	Asteraceae	*Helichrysum* *graveolens* (M.Bieb) Sweet	Afsantin	Aerial parts	In^2^	Anodyne, Anthelmintic, Appetizer, Nerve Tonic
56	Asteraceae	*Lactuca* *sativa* L.	Kahu	Seed	In^1^	Anti-thirst, Hypnotic
57	Asteraceae	*Matricaria chamomilla *L.	Gole babooneh	Flower	In^1^	Eczema, Antitussive, Anticatarrhal, Hair Tonic, Treatment of Colic, Menstrual Pains
58	Asteraceae	Silybum marianum (L.) Gaertn.	Khare Maryam	Seed	In^1^	Jaundice, Febrifuge, Antihepatitis, Liver Tonic
59	Asteraceae	*Tagetes* *erecta* L.	Gol Jafari	Flower	Im^1^	Febrifuge, Treatment of Cut
60	Asteraceae	*Tanacetum parthenium *(L.) Sch. Bip.	Gole babooneh	Flower	In^2^	Antitussive, Anticatarrhal, Hair Tonic, Treatment of Colic, Menstrual Pains
61	Asteraceae	Tripleurospermum disciforme (C. A. Mey.) Sch.Bip.	Gole babooneh	Flower	In^2^	Treatment of Cough, Febrifuge
62	Asteraceae	*Tussilago farfara* L.	Pa Khari	Aerial parts	In^1^	Expectorant, Antitussive, Mouth Wounds, Treatment of Furuncles
63	Berberidaceae	*Berberis integrrima *Bunge	Zereshk Kuhi	Fruit	In^1^	Hypoglycemic, Antihypertensive, Blood and Liver Cleanser, Jaundice, Febrifuge, Antigout
64	Berberidaceae	*Berberis* *sp.*	Zereshk	Fruit	In^1^	Antigout, Blood and Liver Cleanser, Febrifuge, Anthelmintic,Treatment of Dysentery
65	Betulaceae	*Corylus* *avellana* L.	Fandogh	Fruit	In^1^	Treatment of Anaemia, Depurative, Appetizer
66	Boraginaceae	Arnebia euchroma (Royle) I.M.Johnst.	Havachoobeh	Root	In^1^	Treatment of Dermal Disorders, Hair Tonic
67	Boraginaceae	*Caccinia macranthera *(Banks & Sol.) Brand	Gavzaban sabz	Aerial parts	In^1^	Sedative, Treatment of Cough, Expectorant
68	Boraginaceae	*Cordia* *myxa* L.	Sepestan	Fruit	In^1^	Pharyngitis, Antitussive, Febrifuge, Laxative
69	Boraginaceae	Echium amoenum Fisch. & C.A.Mey.	Gole Gavzaban	Flower	In^1^	Antihypertensive, Nerve Tonic, Diuretic, Antistress, Blood Cleanser Cardiac Tonic
70	Boraginaceae	*Trichodesma incanum* (Bunge) A.DC.	Alaf-e- simkesh	Aerial parts	In^1^	Treatment of Bone Fracture
71	Brassicaceae	*Alyssum* *alyssoides *(L.) L.	Ghodumeh	Seed	In^1^	Pharyngitis, Antitussive, Febrifuge, Laxutive, Treatment of Hoarseness
72	Brassicaceae	*Anastatica hierochuntica* L.	Change maryam	Aerial parts	In^1^	Bring Luck to Pregnant Women, Menstrual Regulator
73	Brassicaceae	*Brassica* *napus* L.	Shalgham	Seed	In^1^	Antiseptic, Treatment of Cold, Tonic
74	Brassicaceae	Brassica nigra (L.) K.Koch	Khardal	Seed	In^1^	Laxative
75	Brassicaceae	Capsella bursa-pastoris (L.) Medik.	Kiseh Keshish	Seed	In^1^	Period Regulator, Anti-hemorrhage, Antidiarrhea
76	Brassicaceae	Descurainia sophia (L.) Webb ex Prantl	Khakshir	Seed	In^1^	Blood and Liver Cleanser, Jaundice, Febrifuge, Treatment of Furuncles, Anti-thirst, Laxative
77	Brassicaceae	Eruca sativa (L.) Mill.	Mandab (Roghan cheragh)	Seed	In^1^	Sedative, Laxative Diuretic, Stomach Tonic,
78	Brassicaceae	*Lepidium* *sativum* L.	Shahi (Tartizak)	Seed	In^1^	Appetizer, Anthelmintic, Laxative, Sore Throat
79	Brassicaceae	*Nasturtium officinale *R. Br.	Alafe cheshmeh	Aerial parts	In^1^	Diabetes, Dyspepsia
80	Burseraceae	*Boswellia sacra *Fluek.	Kondor	Gum	Im^1^	Memory Tonic Treatment of Premature Ejaculation,
81	Burseraceae	*Commiphora* *mukul* (Hook. Ex Stocks) Engl.	Moghle Azragh	Gum	Im^1^	Treatment of Joints Pain, Carminative
82	Burseraceae	*Commiphora* *myrrha* (Nees) Engl.	Morre makki	Gum	Im^1^	Obesity, Hematinic,Carminative Antibacterial, Laxative, Stomachache
83	Cannabinaceae	*Cannabis* *sativa* L*.*	Shahdaneh	Seed	In^1^	Sedative, Tonic Treatment of Osteoarthritis, Treatment of Ear Pain
84	Cannabinaceae	*Humulus* *lupulus* L*.*	Razak	Hops	In^1^	Duretic, Treatment of Sleeplessness, Kidney Tonic, Calming, Sedative for Digestion
85	Capparaceae	*Capparis* *spinosa* L*.*	Kavar	Fruit- Root	In^1^	Liver Tonic, Hepatitis, Appetizer, Anthelmintic, Stomach Tonic, Emmenagogue, Antigout
86	Caprifoliaceae	Nardostachys jatamansi (D.Don) DC.	Sonboletib	Root	Im^2^	Nerve Tonic, Cardiac Tonic, Hypnotic, Anti-Stress, Antimigraine, Treatment of Incontinence
87	Caryophylaceae	Acanthophyllum sordidum Bunge ex Boiss.	Choobak	Root	In^1^	Warts, Washing
88	Colchicaceae	*Colchicum* *autumnale* L.	Suranjan	Root	In^1^	Antigout, Calmative, Arthrodynia
89	Combretaceae	Terminalia bellirica (Gaertn.) Roxb.	Balileh	Fruit	Im^1^	Laxative, Appetizer
90	Combretaceae	Terminalia chebula Retz.	Halileh Siah	Fruit	Im^1^	Purgative, Treatment of Constipation, Liver Tonic, Antihemorrhoids
91	Combretaceae	Terminalia citrina Roxb. ex Fleming	Halileh Zard	Fruit	Im^1^	Antidiarrhea, Anthelmintic, Digestive
92	Convolvolaceae	*Cuscuta* *epithymum* Murray	Aftimun	Aerial parts	In^1^	Laxative, Antihemorrhoids
93	Convolvolaceae	Operculina turpethum (L.) Silva Manso	Torbod	Root	Im^1^	Treatment of Osteoarthritis, Duretic, Laxative, Febrifuge
94	Cornaceae	*Cornus mas *L*.*	Zoghal Akhteh	Fruit	In^1^	Prostatichypertropy, Antihemorrhage, Antidiarrhea, Febrifuge
95	Cucurbitaceae	Citrullus colocynthis (L.) Schrad.	Hanzal	Fruit- Seed	In^1^	Purgative, Anodyne, Hypoglycemic
96	Cucurbitaceae	*Cucumis* *sativus* L.	Khiar	Seed	In^1^	Diuretic, Antilithiasis, Blood-cleansing, Febrifuge
97	Cucurbitaceae	*Cucurbita* *pepo* L.	Kadu Tokhm Kaghazi	Seed	Im^1^	Prostatichypertrophy
98	Cupressaceae	*Juniperus* *sabina* L.	Abhal	Fruit	In^1^	Diuretic, Antilithiasis, Food digestion, Urinary Antiseptic
99	Cyperaceae	*Cyperus* *rotundus* L.	Soade Kufi	Root	In^1^	Strengthening of Memory
100	Elaeagnaceae	*Elaeagnus* *angustifolia* L*.*	Senjed	Fruit	In^1^	Arthrodynia, Antidiarrhea, Treatment of Rheumatism, Female Aphrodisiac
101	Ephedraceae	Ephedra major Host	Khakestar Koshtar	Aerial parts	In^1^	Treatment of Joints Pain
102	Equisetaceae	*Equisetum* *arvense* L.	Dome Asb	Aerial parts	In^1^	Obesity, Antilithiasis, Antihypertensive, Prostate Disorders, Treatment of kidney Disorders
103	Ericaceae	Vaccinium arctostaphylos L.	Ghareh Ghat	Fruit	In^1^	Diabetes, Depurative, Antihypertensive, Calmative
104	Euphorbiaceae	*Ricinus* *communis* L.	Karchak	Seed	In^1^	Purgative
105	Fabaceae	*Abrus* *precatorius* L.	Cheshm Khorus	Seed	Im^1^	Contraceptive
106	Fabaceae	Chamaecrista *absus* (L.) H.S. Irwin & Barneby	Cheshom	Seed	Im^1^	Pharyngitis, Antitussive, Obesity, Carminative
107	Fabaceae	Acacia senegal (L.) Willd.	Samgh Arabi	Gum	Im^1^	Cystitis, Antitussive, Gastric ulcer, Anti-inflammatory, Hoarseness
108	Fabaceae	*Alhagi graecorum* Boiss.	Taranjabin	Manna	In^1^	Jaundice, Laxative, Febrifuge, Thirst, Aphthous Ulcers
109	Fabaceae	*Alhagi* *maurorum* Medik.	Khar Shotor- Taranjabin	Aerial parts - Manna	In^1^	Appetite Suppressant, Diuretic, Jaundice, Febrifuge
110	Fabaceae	Astragalus adscendens Boiss. & Hausskn. ex Boiss.	Gazangabin	Manna	In^1^	Laxative, Febrifuge Digestive
111	Fabaceae	Astragalus *fasciculifolius* subsp. *arbusculinus* (Bornm. & Gauba)Tietz	Anzerut	Gum	In^1^	Antitussive, Jaundice, Laxative, Anthelmintic
112	Fabaceae	*Astragalus* *hamosus* L.	Nakhonak	Fruit	In^1^	Anodyne, Repel of Kidney Stone, Diuretic, Arthrodynia, Carminative
113	Fabaceae	*Astragalus* *sieversianus* Pall.	Gol Sefid	Fruit	In^1^	Menstrual Disorders
114	Fabaceae	*Astragalus* *spp.*	Katira	Gum	In^1^	Mouth Wounds, Aphrodisiac, Cystitis, Hair Tonic
115	Fabaceae	*Cassia* *fistula* L.	Folus	Fruit	Im^1^	Treatment of Leishmaniasis, Infant Colic, Febrifuge, Purgative, Jaundice
116	Fabaceae	Entada gigas (L.) Fawc. & Rendle	Ghorse Kamar	Seed	Im^1^	Washing, Aphrodisiac, Hair Tonic
117	Fabaceae	*Glycyrrhiza* *glabra* L*.*	Shirin Bayan	Root	In^1^	Antitussive, Antacid, Tonic, Gastric ulcer, Treatment of Hypotension, Treatment of Anaemia
118	Fabaceae	Indigofera argentea Burm.f.	Rang	Leaves	In^1^	Anti Fungal, Hair color, Hair Tonic
119	Fabaceae	*Lupinus* *luteus* L*.*	Baghelaye Mesri	Seed	Im^1^	Anthelmintic, Emmenagogue, Carminative, Liver Tonic
120	Fabaceae	*Medicago* *sativa* L*.*	Yunjeh	Aerial parts	In^1^	Appetizer, Tonic, Osteomalacia, Anti hemorrhage
121	Fabaceae	*Securigera* *securidaca* (L*.*) Degen & Dorfl.	Gandeh Talkheh	Seed	In^1^	Diabetes, Antihyperlipidemia
122	Fabaceae	Senna *italica* subsp.* italica* Mill.	Sena	Leaves	Im^1^	Purgative, Obesity, Treatment of Hemorrhoids
123	Fabaceae	*Tamarindus* *indica* L*.*	Tamr Hendi	Fruit	Im^1^	Jaundice, Depurative, Pimples
124	Fabaceae	Trigonella foenum-graecum L.	Shanbalileh (Holbeh)	Seed	In^1^	Diabetes,Bronchitis, Osteomalacia, Antihyperlipidemia, Tonic, Treatment of Anaemia
125	Fagaceae	Quercus infectoria Oliv.	Mazuye sabz	Insect gull	In^1^	Nose-Bleed, Anti-Hemorrhage, Uterus Aailments, Mouth Wounds, Antihemorrhoids
126	Fagaceae	*Quercus* *spp.*	Balut (Mazu)	Fruit	In^1^	Antidiarrhea, Anti-hemorrhage
127	Gentianaceae	*Gentiana* *lutea* L.	Jentiana	Root	Im^1^	Aphrodisiac, Blood Tonic
128	Gentianaceae	Gentiana olivieri Griseb.	Suloo	Flower	In^1^	Cardiac Ailments
129	Grossulariaceae	Ribes khorasanicum Saghafi & Assadi	Ghareh Ghat	Fruit	In^2^	Antihypertensive, Diabetes, Depurative
130	Hypericaceae	*Hypericum* *scabrum* L*.*	Hufarighun	Flower	In^1^	Antimigraine, Gastric ulcer, Aanti hemorrhage, Urinary Incontinence, Treatment of Headache
131	Iridaceae	*Crocus* *sativus* L*.*	Zaffaron	Style	In^1^	Tonic, Dysmenorrheal, Emmenagogue, Nerve Tonic, Premature ejaculation, Gastric ulcer, Aphrodisiac
132	Iridaceae	*Iris* *spuria *L.	Zanbagh	Root	In	Arthrodynia, Diuretic
133	Juglandaceae	*Juglans* *regia* L*.*	Gerdu	Fruit- Leaves	In^1^	Eczema, Antidiarrhea, Hair color
134	Lamiaceae	Clinopodium *graveolens* (M.Bieb.) Kuntze	Faranjmeshk	Seed	In^1^	Pharyngitis, Gastric Ulcer, Nerve Tonic
135	Lamiaceae	*Hymenocrater* *spp.*	Badranjbuyeh	Aerial parts	In^2^	Cardiac Tonic, Hypnotic, Antitussive, Carminative, Dyspnoea, Anti-stress Convulsion
136	Lamiaceae	Hyssopus officinalis L.	Zufa	Aerial parts	Im^1^	Pulmonary Infections, Treatment of Cold, Expectorant
137	Lamiaceae	Lallemantia iberica (M.Bieb.)Fisch. & C.A.Mey.	Tokhm Sharbati	Seed	In^1^	Gastric Ulcer, Antitussive, Laxative, Hoarseness, Anti-thirst
138	Lamiaceae	Lavandula angustifolia Mill.	Lavand	Aerial parts	Im^1^	Treatment of Insomnia, Treatment of cold, Nerve Tonic, Cardiac Tonic
139	Lamiaceae	*Marrubium vulgar*e L*.*	Ferasion	Aerial parts	In^1^	Liver Tonic, Antitussive
140	Lamiaceae	Melissa officinalis L.	Badranjbuyeh	Aerial parts	In^1^	Nerve Tonic, Cardiac Tonic, Hypnotic, Antitussive, Carminative, Anti-tress, Convulsion
141	Lamiaceae	*Mentha longifolia* (L.) Hudson	Puneh	Aerial parts	In^1^	Herpes, Anthelmintic, Antacid, Carminative, Antidiarrhea, Digestive
142	Lamiaceae	*Mentha* *spicata* L.	Naana	Aerial parts	In^1^	Appetizer, Antacid, Carminative, Antidiarrhea, Digestive, Anodyne ,Anthelmintic
143	Lamiaceae	*Nepeta binaloudensis* Jamzad	Ostokhodus	Aerial parts	In^2^	Treatment of Cold, Carminative, Nerve Tonic, Treatment of Sinusitis, Pulmonary infections, Treatment of Rheumatism, Antiasthmatic, Aantitussive, Cardiac Tonic
144	Lamiaceae	Nepeta bracteata Benth.	Zufa	Aerial parts	In^2^	Pulmonary Infections, Antiasthmatic, Treatment of cold, Febrifuge, Treatment of Colic, Antitussive
145	Lamiaceae	Nepeta menthoides Boiss. & Buhse	Ostokhodus	Aerial parts	In^1^	Treatment of Cold, Nerve Tonic, Expectorant
146	Lamiaceae	Ocimum basilicum L.	Reyhan (Tokhm sharbati)	Seed	In^1^	Aphthous Ulcers, Antiseptic, Antidiarrhea, Antitussive, Carminative, Laxative, Digestive, Antacid
147	Lamiaceae	*Origanum* *vulgare* L.	Marzanjush	Aerial parts	In^1^	Treatment of Colic, Treatment of Sinusitis, Sedative, Cardiac Tonic, Nerve Tonic, Treatment of Dyspnoea
148	Lamiaceae	Perovskia abrotanoides Kar.	Gol Kabud	Aerial parts	In^1^	Treatment of Sinusitis, Treatment of Toothache, Antitussive, Nerve Tonic, Carminative, Sedative, Antiseptic, Anthelmintic, Treatment of Colic
149	Lamiaceae	*Rosmarinus* *officinalis* L.	Rozmari	Leaves- Flower	Im^1^	Treatment of Joints Pain, Hair Loss, Depression, Nerve Tonic, Appetizer, Hypnotic
150	Lamiaceae	Salvia leriifolia Benth.	Noruzak	Aerial parts	In^1^	Diabetes, Period Regulator
151	Lamiaceae	Salvia macrosiphon Boiss.	Kenocheh	Seed	In^1^	Jaundice, Antitussive, Febrifuge , Gastric ulcer, Pharyngitis, Laxative
152	Lamiaceae	*Salvia* *officinalis* L.	Maryam Goli	Aerial parts	Im^1^	Female Fertility, Hypoglycemic, Menopause, Stomach Tonic
153	Lamiaceae	*Satureja* *hortensis* L.	Marzeh	Aerial parts	In^1^	Indigestion, Anthelmintic, Appetizer, Antacid, Antidiarrhea
154	Lamiaceae	Stachys lavandulifolia Vahl	Chai Kuhi	Flower	In^1^	Nerve Tonic, Treatment of cold, Cardiac Tonic, Treatment of Colic
155	Lamiaceae	*Teucrium* *polium* L*.*	Kalpureh	Aerial parts	In^1^	Antacid, Indigestion, Diabetes, Treatment of Colic, Antidiarrhea
156	Lamiaceae	*Vitex* *negundo* L*.*	Felfel Kuhi	Fruit	In^1^	Menstrual regulator, Obesity, Treatment of Sinusitis
157	Lamiaceae	Zataria multiflora Boiss.	Avishan Shirazi	Aerial parts	In^1^	Treatment of Sinusitis, Menstrual Pains, Dysmenorrheal, Anthelmintic, Antacid, Treatment of Colic, Antiasthmatic, Dyspnoea, Arthrodynia, Carminative
158	Lamiaceae	Ziziphora clinopodioides Lam.	Avishan kuhi	Aerial parts	In^1^	Kidney Pain, Antacid, Carminative,Treatment of Colic, Anthelmintic, Antitussive, Antidiarrhea, Digestive
159	Lamiaceae	*Ziziphora* *teniuor* L.	Kakuti	Aerial parts	In^1^	Digestive, Treatment of Colic, Calefacient, Antacid, Antiseptic
160	Lauraceae	*Cinnamomum camphora* (L.) J. Presl	Kafur	Gum	Im^1^	Antiaphrodisiac, Anodyne, Treatment of Toothache
161	Lauraceae	*Cinnamomum* *zeylanicum* Nees	Darchin	Bark	Im^1^	Treatment of Headache, Calmative, Urinary Incontinence, Digestive, Flavor, Hypoglycemic, Carminative
162	Lauraceae	*Laurus* *nobilis* L.	Barg Bu	Leaves	In^1^	Carminative Appetizer, Flavor
163	Liliaceae	*Fritillaria* *imperialis* L.	Laleh Sarnegun	Root	In^1^	Treatment of Joints Pain
164	Linaceae	*Linum* *usitatissimum* L.	Katan	Seed	In^1^	Cholesterol Lowering, Antitussive, Laxative, Obesity
165	Loganiaceae	Strychnos nux-vomica L.	Kachuleh	Seed	Im^1^	Antiallergic, Eczema, Sedative
166	Lythraceae	*Lawsonia* *inermis* L.	Hana	Leaves	In^1^	Hair color Treatment of headache, Hair Tonic, Washing, , Anti Fungal, Antiseptic
167	Malvaceae	*Abelmoschus esculentus *(L.) Moench	Bamieh	Seed	In^1^	Anti-inflammatory, Diuretic, Laxative
168	Malvaceae	*Alcea* *spp.*	Gole Khatmi	Flower	In^1^	Antitussive, Febrifuge, Treatment of pimples, Laxative, Depurative, Treatment of gum swelling
169	Malvaceae	*Althaea* *officinalis* L*.*	Charme giah	Root	In^1^	Mouth Wounds, Bone Fracture, Treatment of Bruises, Treatment of Dysuria
170	Malvaceae	*Helicteres* *isora* L*.*	Bahman pich	Fruit	Im^1^	Antidiarrhea, Antispasmodic
171	Malvaceae	*Hibiscus* *sabdariffa* L.	Chai Makkeh	Flower	Im^1^	Hypoglycemic, Antihypertensive, Obesity, Blood-cleansing, Calmative, Cardiac Tonic
172	Malvaceae	*Hibiscus syriacus *L.	Gole Khatmi	Flower	In^2^	Febrifuge, Antitussive
173	Malvaceae	Malva neglecta Wallr.	Nan Kalagh	Flower- Fruit	In^1^	Sore Throat, Antitussive, Febrifuge
174	Malvaceae	*Malva* *sylvestris* L*.*	Panirak (Khatmi khabbzi)	Flower- Fruit	In^1^	Pharyngitis, Furuncles, Aphthous Ulcers , Febrifuge, Antitussive, Jaundice, Laxative, Gastric ulcer, Treatment of Wounds
175	Malvaceae	*Theobroma* *cacao* L*.*	Kakao	Fruit	Im^1^	Tonic, Flavoring
176	Malvaceae	Tilia cordata Mill.	Zirfun	Leaves- Fruit	In^1^	Nerve Tonic, Sudorific, Duretic, Calmative
177	Menispermaceae	Anamirta cocculus (L.) Wight & Arn.	Mass Mahi	Fruit	Im^1^	Fishering
178	Moraceae	*Ficus* *carica* L*.*	Anjir	Fruit	In^1^	Anti-hemorrhoids, Laxative, Tonic
179	Moraceae	*Morus* *nigra* L*.*	Shatut	Root	In^1^	Abortion
180	Myristicaceae	Myristica fragrans Houtt.	Joze Buya	Seed	Im^1^	Anti-Hemorrhage, Tonic, Appetizer, Treatment of Colic
181	Myrtaceae	*Eucalyptus* *sp.*	Okaliptus	Leaves- Fruit	Im^1^	Treatment of Sinusitis, Treatment of Cold, Treatment of Headache
182	Myrtaceae	*Myrtus* *communis* L*.*	Murd	Leaves- Fruit	In^1^	Psoriasis, Treatment of Sinusitis, Mouth Ulcers, Anti Fungal, Treatment of Cold, Strengthening of Hair, Herpes
183	Myrtaceae	Syzygium aromaticum (L.) Merr. & L.M.Perry	Mikhak	Flower	Im^1^	Toothache, Antiseptic, Digestive, Aphrodisiac, Carminative, Stimulant
184	Nitrariaceae	*Peganum* *harmala* L*.*	Espand	Seed	In^1^	Diabetes, Antiseptic,Hypnotic, Treatment of Rheumatism and Sciatica Disorders, Anthelmintic, Emmenagogue
185	Nymphaeaceae	*Nymphaea* *alba* L*.*	Nilufar Abi	Flower	In^1^	Expectorant, Hypnotic, Antitussive, Calmative
186	Oleaceae	*Fraxinus* *excelsior* L*.*	Zaban Gonjeshk	Fruit	In^1^	Aphrodisiac, Treatment of Stammering
187	Oleaceae	*Olea* *europaea* L*.*	Zeytun	Fruit- Leaves	Im^1^	Anti-hemorrhoids, Antihyperlipidemia, Hypoglycemic, Laxative, Treatment of Dermal Allergic
188	Orchidaceae	*Anacamptis morio *(L.) R. M. Bateman	Saalab gholveh	Root	In^1^	Tonic
189	Orchidaceae	*Dactylorhiza* *umbrosa* (Kar. & Kir.) Nevski	Saalab panjeh	Root	In^1^	Treatment of Sexual Impotency, Tonic
190	Orchidaceae	*Orchis* *morio* L*.*	Buzidan	Root	Im^1^	Cardiac Tonic, Arthrodynia, Tonic, Hypnotic, Insecticide
191	Orchidaceae	Vanilla planifolia Jacks. ex Andrews	Vanil	Fruit	Im^1^	Stimulant, Tonic, Carminative, Flavoring
192	Paeoniaceae	*Paeonia* *officinalis* L*.*	Ude Salib	Root	Im^1^	Antiepileptic, Carminative, Treatment of Colic
193	Papaveraceae	*Fumaria* *vaillantii* Loisel.	Shatareh	Aerial parts	In^1^	Pimples, Febrifuge, Blood-cleansing Psoriasis, Appetizer, Antiacid, Jaundice,
194	Papaveraceae	*Papaver rhoeas* L*.*	Shaghayegh	Flower	In^1^	Treatment of Addiction , Calmative, Sleeplessness, Sedative, Expectorant, Antitussive, Antiasthmatic
195	Papaveraceae	*Papaver* *somniferum* L*.*	Khashkhash	Fruit- Seed	In^1^	Anodyne, Laxative, Tonic, Hypnotic
196	Passifloraceae	*Passiflora* *caerulea* L*.*	Gol Saati	Flower	Im^1^	Treatment of Insomnia, Anti-Stress, Calmative
197	Pedaliaceae	*Sesamum* *indicum* L*.*	Konjed	Seed	In^1^	Blood Tonic, Hair Loss, Strengthening of Memory, Increase Sperm Count, Treatment of Skin’s Split, Laxative
198	Phyllanthaceae	*Phyllanthus* *emblica* L.	Agheleh Moghashar	Fruit	Im^1^	Jaundice, Forgetfulness, Cardiac Tonic Appetizer, Hair Tonic,
199	Pinaceae	Pinus gerardiana Wall. ex D.Don	Chalghooz	Fruit	Im^1^	Aphrodisiac, Blood Tonic
200	Piperaceae	*Piper* *cubeba* L*.*	Kababeh Chini	Fruit	Im^1^	Appetizer, Tonic, Carminative
201	Piperaceae	*Piper* *longum* L*.*	Darfelfel	Fruit	Im^1^	Carminative, Tonic, Food Digestion
202	Piperaceae	*Piper* *nigrum* L*.*	Felfel Siah	Fruit	Im^1^	Stimulant, Tonic, Appetizer, Falvoring
203	Plantaginaceae	*Plantago* *major* L*.*	Barhang	Seed- Leaves	In^1^	Eczema, Antiallergic, Febrifuge, Jaundice, Antitussive, Antidiarrhea, ToothacheDepurative, Gastric ulcer
204	Plantaginaceae	Plantago ovata Forssk.	Esfarzeh	Seed	In^1^	Obesity, Depilator, Tonsillitis, Antacid, Antitussive, Gastric ulcer, Febrifuge, Laxative, Jaundice, Antihemorrhoids
205	Platanaceae	Platanus orientalis L.	Chenar	Fruit	In^1^	Prostate Diseases
206	Poaceae	*Arundo* *donax* L*.*	Tabashir ghalam	Latex	In^1^	Aphthous Ulcer, Anti Thirst, Depurative, Treatment of Pimples, Febrifuge
207	Poaceae	Avena sativa L.	Jo dosar	Seed	In^1^	Treatment of Acne
208	Poaceae	*Oryza* *sativa* L*.*	Chaltooke Berenj	Seed coat	In^1^	Hair Tonic, Treatment of Anaemia
209	Poaceae	*Zea mays* L*.*	Kakole Zorat	Style	In^1^	Obesity, Anti-inflammatory, Aantilithiasis, Kidney Disorders, Prostate Disorders, Duretic
210	Polygonaceae	*Persicaria bistorta *(L.) Samp.	Anjebar	Root	Im^1^	Antidiarrhea, Anti-hemorrhage, Vulnerary
211	Polygonaceae	*Polygonum* *aviculare* L*.*	Alaf Haftband	Aerial parts	In^1^	Diabetes, Treatment of Colic, Antidiarrhea
212	Polygonaceae	*Rheum* *palmatum* L*.*	Rivand Chini	Root	Im^1^	Liver Diseases, Cardiac Tonic, Antilithiasis, Backache, Appetizer, Purgative
213	Polygonaceae	*Rheum* *ribes *L.	Rivas	Fruit- Petiole	In^1^	Jaundice, Urinary Antiseptic, Diuretic, Depurative, Liver Tonic, Antiseptic, Hair Tonic
214	Polygonaceae	Rheum turkestanicum Janisch.	Eshghan	Root	In^1^	Diabetes, Antihypertensive, Anticancer, Depurative
215	Polygonaceae	*Rumex* *acetosella* L*.*	Sagh Torshak	Root	In^1^	Jaundice, Febrifuge
216	Polypodiaceae	*Polypodium* *vulgare* L*.*	Baspayak	Root	In^1^	Expectorant, Jaundice, Digestive
217	Polyporaceae	*Polyporus* *officinalis* Fries	Gharighun	Fruiting body	Im^1^	Anti-hemorrhage, Laxative
218	Portulacaceae	*Portulaca* *oleracea* L.	Khorfeh	Seed- Leaves	In^1^	Antitussive, Febrifuge, Anti-Thirst, Food digestion, Depurative, Duretic, Anti-hemorrhoids
219	Primulaceae	Embelia ribes Burm.f.	Barang Kaboli	Fruit	Im^1^	Jaundice, Anthelmintic, Antidiarrhea
220	Pteridaceae	Adiantum capillus-veneris L.	Parsiavashan	Aerial parts	In^1^	Antitussive, Antihemorrhoid, Treatment of Sore Throat, Febrifuge, Jaundice, Laxative, Antithirst, Treatment of Orchitis,
221	Punicaceae	*Punica* *granatum *L*.*	Gole Anar	Flower- Root	In^1^	Anti-hemorrhage, Blood flux, Anthelmintic
222	Ranunculaceae	Delphinium semibarbatum Bien. ex Boiss.	Zarir	Flower	In^1^	Treatment of Dermal Allergies, Coloring
223	Ranunculaceae	*Nigella* *sativa* L*.*	Siah Daneh	Seed	In^1^	Kidney Stone, Carminative, Antacid, Galactogogue, Anthelmintic, Food Digestion, Antitussive, Treatment of Colic
224	Ranunculaceae	*Thalictrum sultanabadense *Stapf	Parsiavashan	Aerial parts	In^2^	Antitussive, Febrifuge
225	Rhamnaceae	*Ziziphus* *jujuba *Miller	Annab	Fruit	In^1^	Depurative, Febrifuge, Laxative, Jaundice, Antitussive, Treatment of Thirst
226	Rhamnaceae	Ziziphus spina-christi (L.) Willd.	Sedr	Leaves	In^1^	Eczema, Hair Tonic, Anti Fungal, Antipruritic, Washing
227	Rosaceae	Cerasus avium (L.) Moench	Dome Gilas	Pedicel	In^1^	Antilithiasis, Prostate Disorders Kidney Stone, Anti-inflammatory,
228	Rosaceae	*Crataegus* *sp.*	Sorkhe Valik	Fruit- Leaves	In^1^	Depurative, Repairs Blood Vessel
229	Rosaceae	Cotoneaster nummularius Fisch. & C.A.Mey.	Shir Khesht	Manna	Im^1^	Jaundice, Febrifuge
230	Rosaceae	*Cydonia* *oblonga* Mill.	Beh Daneh	Seed- Leaves	In^1^	Cardiac Diseases, Antitussive, Sore Throat, Laxative, Febrifuge
231	Rosaceae	*Rosa beggeriana *Schrenk	Nastaran	Fruit	In^1^	Antihypertensive, Diuretic, Kidney Stone
232	Rosaceae	Rosa damascena Mill.	Gole Mohammadi	Flower	In^1^	Antihemorrhoid, Laxative, Calmative
233	Rosaceae	Rosa foetida Herrm.	Gole Zard	Flower	In^1^	Ovary Tonic, Emmenagogue
234	Rubiaceae	*Coffea* *arabica* L*.*	Ghahveh	Seed	Im^1^	Obesity, Sleeplessness, Antidiarrhea
235	Rubiaceae	*Cinchona* *officinalis* L*.*	Ganeh Ganeh	Bark	Im^1^	Treatment of Joints Pain, Febrifuge, Antimalaria
236	Rubiaceae	*Rubia* *tinctorum* L*.*	Ronas	Root	In^1^	Strengthening of Hair, Hair color
237	Rutaceae	Citrus aurantiifolia (Christm.) Swingle	Limu Amani	Fruit	In^1^	Antihypertensive, Calmative
238	Rutaceae	Citrus aurantium L.	Bahar Naranj	Flower	In^1^	Anti-Stress, Cardiac Tonic, Food Digestion, Antihypertensive
239	Rutaceae	*Ruta* *graveolens* L*.*	Sodab	Aerial parts	In^1^	Abortion, Sedative, Emmenagogue
240	Rutaceae	*Toddalia* *asiatica* (L.) Lam*.*	Dahan baz-Dahan basteh	Fruit	Im^1^	Diabetes, Febrifuge, Blood flux
241	Salicaceae	*Salix* *alba* L*.*	Bid	Leaves- Bark	In^1^	Menstrual Pains, Anodyne, Jaundice, Antitussive
242	Salicaceae	*Salix* *aegyptiaca* L*.*	Bidmeshk	Flower	In^1^	Calmative, Cardiac Tonic, Painful Menstruation
243	Salicaceae	Salix excelsa J.F.Gmel.	Bidkhesht	Manna	In^1^	Febrifuge, Jaundice, Laxative
244	Santalaceae	*Santalum* *album* L*.*	Sandal	Wood	Im^1^	Aromatic, Duretic Antidiarrhea,
245	Schisandraceae	Illicium verum Hook.f.	Badian Khatai	Fruit	Im^1^	Carminative, Galactogogue, Appetizer
246	Scrophulariaceae	*Digitalis* *purpurea* L*.*	Gol Angoshtaneh	Flower	Im^1^	Cardiac Tonic
247	Scrophulariaceae	*Scrophularia* *striata* Boiss.	Mokhallaseh	Aerial parts	In^1^	Kidney Troubles, Antidiarrhea, Treatment of Colic, Carminative, Treatment of Joints Pain
248	Scrophulariaceae	Verbascum cheiranthifolium Boiss.	Dome Gav	Aerial parts	In^1^	Dyspepsia, Antidiarrhea, Expectorant, Antiacid, Stomach Tonic
249	Solanaceae	Atropa belladonna L.	Beladon	Leaves	In^1^	Antispasmodic, Sedative
250	Solanaceae	*Capsicum* *annuum* L*.*	Felfel Ghermez	Fruit	In^1^	Appetizer, Spice, Treatment of Osteoarthritis, Tonic, Stimulant, Aphrodisiac
251	Solanaceae	*Datura* *stramonium* L*.*	Tatureh	Seed	In^1^	Sedative, Treatment of Addiction, Treatment of Colic
252	Solanaceae	*Hyoscyamus* *niger* L*.*	Bangdaneh	Seed	In^1^	Sedative, Treatment of Addiction, Treatment of Toothache, Treatment of Headache, Antigout
253	Solanaceae	*Physalis* *alkekengi* L.	Arusak Posht Pardeh	Fruit	In^1^	Emmenagogue Treatment of Kidney Stones, Blood Cleanesing,
254	Solanaceae	*Solanum americanum *Mill.	Tajrizi	Fruit	In^1^	Treatment of Osteoarthritis, Mastitis, Expectorant, Hypnotic, Sedative, Treatment of Gastritis
255	Theaceae	Camellia sinensis (L.) Kuntze	Chai Sabz	Leaves	In^1^	Obesity, Anticancer, Antihypertensive, Hepatitis, Antihyperlipidemia
256	Urticaceae	*Urtica* *dioica* L*.*	Gazaneh	Whole plant	In^1^	Hypoglycemic, Enlarged Prostate, Anaemia, Anti-inflammatory, Digestive
257	Urticaceae	Urtica pilulifera L.	Anjareh	Seed	In^1^	Laxative, Treatment of Cough
258	Verbenaceae	Aloysia *citriodora* Palau	Beh Limu	Leaves	Im^1^	Dyspepsia, Nerve Tonic, Appetizer, Carminative, Calmative
259	Verbenaceae	*Verbena officinalis *L*.*	Shahpasand	Aerial parts	In^1^	Appetizer, Indigestion
260	Violaceae	Viola odorata L.	Banafsheh	Flower	In^1^	Eczema, Febrifuge, Antiallergic, Blood Cleansing, Jaundice, Treatment of Cold, Expectorant
261	Xanthorrhoeaceae	Eremurus spectabilis M.Bieb.	Serish	Root	In^1^	Dermal infection, Sticking, Antihyperlipidemia
262	Zingiberaceae	Alpinia galanga Willd.	Ghost Shirin	Root	Im^1^	Treatment of Bloody Diarrhea, Treatment of Osteoarthritis
263	Zingiberaceae	Alpinia officinarum Hance	Kholanjan	Root	Im^1^	Treatment of Rheumatism, Tonic, Carminative, Digestive, Aphorodisiac
264	Zingiberaceae	Amomum subulatum Roxb.	Hele bad	Fruit	Im^1^	Carminative, Flavoring, Treatment of Colic, Flavoring
265	Zingiberaceae	*Curcuma* *longa* L*.*	Zard Chubeh	Root	Im^1^	Treatment of Gall Stones, Treatment of Bruises, Digestive, Emmenagogue
266	Zingiberaceae	Curcuma zedoaria (Christm.) Roscoe	Zorombad	Root	Im^1^	Liver Tonic, Carminative, Tonic, Indigestion , Obesity, Flavoring
267	Zingiberaceae	Elettaria cardamomum Maton	Hel	Fruit	Im^1^	Flavoring, Treatment of Flatulence, Appetizer, Tonic
268	Zingiberaceae	Zingiber officinale Roscoe	Zanjafil	Root	Im^1^	Increase of Sperm Count , Obesity, Carminative, Tonic, Flavoring, Treatment of Joints Pain, Treatment of Toothache
269	Zygophyllaceae	*Tribulus* *terrestris* L*.*	Kharkhasak	Aerial parts	In^1^	Duretic, Kidney Stone, Tonic, Treatment of Prostate Hypertrophy, Anthelmintic, Jaundice, Treatment of Flooding, Treatment of Dysuria, Urinary Antiseptic

**Status**: (**Im**: Imported sample, **In**: Indigenous sample, **1**: Authentic sample, **2**: Adulterated or Substituted sample).

## Discussion

The survey indicated that the study area has plenty of medicinal plants to treat a wide spectrum of human ailments. Herbal medicine uses reported by herbalists can be classified into 132 different uses. Traditional knowledge of phytotherapy of this region provides excellent results in the treatment of jaundice, diarrhea, kidney stones, eczema, obesity, psoriasis, arthritis, diabetes, bone fracture, stomachache, cancer, migraine, joints pains, asthma, headache, skin problems, urinary troubles, wound, toothache, purification of blood, constipation, intestinal worms, pimples, and many other ailments. The highest number of species and applications were reported for digestive system disorders (38.4 %). Significant results are noticed in terms of treatments of respiratory system disorders such as sinusitis, asthma, and bronchitis. 

The most efficient medicinal plants are *Perovskia abrotanoides, Dysphania botrys, Coriandrum* *sativum, Vitex* *negundo,* *and Ziziphora clinopodioides*. Diseases such as jaundice, diarrhea, anemia, rheumatism, joints pains, and dysmenorrheal were obviously easily diagnosed by the old herbalists. General physical condition and tongue or eyes color of the patient are used as indicators of the patient’s problem. Through the analysis of records of plant-based medicinal treatments (Table 1), it becomes obvious that some of the plants are being used more frequently than others (*Achillea santolinoides, Astragalus* *gummifer, Bunium persicum, Cichorium* *intybus, Echium amoenum, Glycyrrhiza* *glabra, Malva* *sylvestris,* *Nardostachys jatamansi, Plantago ovate, and Ziziphora clinopodioides*). Aerial parts (stem, leaves, and flowers) are the most frequently used parts in medicinal purposes. Present investigation showed that the traditional drugs sold in herbal shops in the city of Mashhad may be adulterated or substituted with quite unrelated plant materials. Due to some morphological similarities of the plant parts and their improper identification by the consumers and herbal plant sellers and lack of a standard identification system, the crude medicinal plants and their parts are often adulterated or substituted in commerce which may result in the loss of their efficacy. For instance, *Alcea* spp. especially (*Alcea rosea*, *Alcea aucheri*, *Alcea angulata, Alcea rhyticarpa, *and *Alcea lavateriflora*) are known as Gol-e-khatmi in different parts of study area and* Hibiscus syriacus *L. adulterated or substituted instead of them and also *Thalictrum sultanabadense *Stapf are sold instead of Adiantum capillus-veneris L. in some market samples. 

Distinction and identification of medicinal plants are very important because the adulterants, although belonging to the same genus as the drug, does not possess the medicinal properties of the drug. For example, *Bunium cylindricum *are mixed with real Zire-e-siah (*Bunium persicum*) and are sold in the market resulting in the degrading of the quality and efficacy of the drug. Correct identification of herbal drugs is the foundation of safe use of herbal medicines and products. Therefore, in order to ensure safety, therapeutic potency and efficacy of herbal medicines, correct identification, authentication, and elimination of adulteration are essential and the drugs should only be authenticated by a panel of experts including taxonomists (Joharchi and Amiri, 2012 ▶). 

This investigation indicated that 269 medicinal plant species belonging to 88 families were found in the research area. Among them, 193 species were indigenous of Iran and 76 species were imported from other countries. These plants are used in the treatment of many diseases. Market survey revealed that there are more than 600 herbalist shops trading natural medicinal products in the Mashhad markets. By comparing present applications of medicinal plants with available literature reported from other regions of Iran, it appears that there are many medicinal uses for the treatment of various ailments in the study area which were rarely demonstrated before this. To our knowledge, the use of *Anastatica hierochuntica,* *Gentiana olivieri, Helichrysum* *graveolens, Mangifera indica, Platanus orientalis, Rheum turkestanicum, Strychnos nux-vomica, *and *Trichodesma incanum* to cure different illnesses, have never been reported before. Further research should be carried out in the field of pharmacology, phytochemistry, and biotechnology of these resources which may lead to the development of new plant-based medicines. From this study, it is concluded that although the people in research area have access to modern medical facilities, a lot of them still use traditional medicine for their healthcare problems. This represents a medicinal alternative for healing health problems which remains closer to the cultural and social context of this society.
